# Investigating the association of bioelectrical impedance analysis–derived phase angle and depressive symptoms in patients receiving maintenance hemodialysis: a cross-sectional study

**DOI:** 10.3389/fnut.2025.1662484

**Published:** 2025-09-09

**Authors:** Lin Huang, Yan Zhang, Jinbao Wang, Jiajun Zhou

**Affiliations:** ^1^Blood Purification Center, Affiliated Yijishan Hospital of Wannan Medical College, Wuhu, China; ^2^Department of Nephrology, The Second Affiliated Hospital of Anhui Medical University, Hefei, China

**Keywords:** bioelectrical impedance analysis, phase angle, depressive symptoms, maintenance hemodialysis, association

## Abstract

**Background:**

Depressive symptoms are prevalent among patients undergoing maintenance hemodialysis (MHD), yet its association with phase angle (PhA, a biomarker of nutritional status and cellular integrity) remains unexplored at present. This study aims to investigate the relationship between PhA value and depression in MHD patients.

**Methods:**

This cross-sectional study was performed with 369 MHD patients in China. PhA was measured using bioelectrical impedance analysis, and depressive symptoms (with score ≥ 10) were assessed using the Patient Health Questionnaire. The associations of PhA and depression were assessed using Spearman rank correlation analysis and unadjusted/adjusted binary logistic regression models, controlling for various clinical, biochemical, and demographic factors.

**Results:**

Among the 369 patients, 17.10% patients (*n* = 63) had depression with 47.60% of male and median age of 58.50 years. There was a significant decrease in the prevalence of depressive symptoms in patients with the increasing quartiles of PhA levels. Compared patients in Q3 and Q4, MHD patients in the lower quartile groups of PhA (Q1 and Q2) exhibited significantly (*p* < 0.001) higher cognitive-affective scores and somatic scores. The Spearman correlation and logistic regression analyses revealed the potential associations between depression and several factors, including comorbidities, albumin, PhA, skeletal muscle index, fat free mass, total body water, and extracellular water/total body water (*p* < 0.05). The unadjusted analysis suggested a significant association between lower PhA values (Q1 and Q2) and higher risk of depressive symptoms. After adjusting for age, sex, and duration of hemodialysis (Model 2), this relationship remained significant for Q1 (OR = 6.051, *p* < 0.001) and Q2 (OR = 3.309, *p* = 0.020). In the fully adjusted model (Model 4), the association between low PhA and depressive symptoms was even more pronounced, with the odds of depression in Q1 reaching an OR of 51.760 (*p* = 0.003).

**Conclusion:**

Lower PhA values were independently and significantly associated with depressive symptoms in MHD patients, suggesting that PhA might serve as an important biomarker for identifying individuals at higher risk for depression. Interventions aimed at improving nutritional status and muscle mass in these patients can potentially reduce the risk of mental deterioration.

## 1 Introduction

Chronic kidney disease (CKD) is a global health concern, characterized by sustained kidney damage or dysfunction lasting over 3 months (1, 2). CKD is typically divided into five stages according to the glomerular filtration rate (GFR), with Stage 5 representing a GFR of less than 15 mL/min, which corresponds to end-stage renal disease (ESRD). For ESRD patients, renal replacement therapies such as kidney transplantation, peritoneal dialysis, and hemodialysis are primary treatment options (3). However, patients undergoing maintenance hemodialysis (MHD) often encounter with various psychological complications, including depression, cognitive dysfunction, malnutrition, and sarcopenia (4). Depression is a particularly common psychiatric problem with an estimated prevalence of 21–27% in non-dialysis CKD patients, and the prevalence is even higher among MHD individuals with 23–42% reported in the United States and Europe (5, 6). MHD patients suffering from depression typically exhibit adverse clinical outcomes, including poorer nutritional status, treatment adherence, and quality of life, along with higher hospitalization and mortality rates, making it a significant challenge in managing this patient population (7). Despite its high prevalence, depression in MHD patients is often undiagnosed, underdiagnosed, or inadequately treated due to the lack of objective and quantifiable indicators in clinical practice. The complex relationship between depressive symptoms, comorbid conditions, and psychosocial factors necessitates a comprehensive approach to manage psychosomatic disorders in MHD patients (8). Early identification and timely interventions of depressive symptoms are critical for these patients to improve their clinical outcomes and quality of life. Additionally, patients with depressive symptoms have been reported to be closely associated with malnutrition, sarcopenia, and poor physical activity (9). These findings underscore the importance of body composition as a potential approach for investigating and monitoring depression in MHD patients. Therefore, understanding the potential relationship between body composition and depression in patients with MHD may lead to more effective strategies for managing depression, ultimately contributing to better clinical outcomes for this vulnerable patient group.

Bioelectrical impedance analysis (BIA) is a non-invasive, rapid, and reliable method widely validated for evaluating hydration status and body composition (10). Its application in hemodialysis patients has become increasingly prevalent, particularly for managing dry weight and nutritional status. The phase angle (PhA) obtained from BIA represents the relationship between body resistance and reactance in response to an applied external electrical current (11). As the most clinically significant impedance parameter, PhA serves as an indicator of cellular membrane integrity and overall cell viability (12). Unlike other BIA-derived measures, PhA is a direct measurement and thus less susceptible to errors arising from assumptions inherent in body composition or hydration estimations. Lower PhA values are indicative of compromised cell membrane integrity or increased cellular apoptosis, whereas higher values reflect a greater proportion of intact and healthy cell membranes (13). Additionally, PhA has recently emerged as a valuable biomarker for monitoring disease progression and predicting clinical outcomes across various medical conditions, such as depressive symptoms, cognitive dysfunction, and sarcopenia (14).

However, to the best of our knowledge, there is still limited information regarding the association between PhA and depressive symptoms in patients. Especially, it is rarely seen in the study on the hemodialysis patients in China. Therefore, the aim of our study is to explore the relationship between PhA and depressive symptoms, particularly in MHD patients. We observe that PhA, as a marker of cellular membrane integrity and overall nutritional status, has been associated with depression in some chronic disease populations. Since MHD patients often face nutritional deficiencies, chronic inflammation, and mental health challenges, investigating the link between PhA and depressive symptoms is of significant clinical relevance for this patient group. Based on the described background, we propose the hypothesis that lower PhA may be correlated with more severe depressive symptoms. This research is expected to investigate the complex factors influencing depression in patients undergoing hemodialysis, with the goal of accelerating the fast diagnosis of depression in this group using PhA as a biomarker. By examining these factors, we hope to provide clinicians with a new tool to better assess and manage depressive symptoms in MHD patients, ultimately leading to improved overall health outcomes for these individuals.

## 2 Patients and methods

### 2.1 Study subjects

A single-center cross-sectional study was conducted at Affiliated Yijishan Hospital of Wannan Medical College from February 2025 to July 2025. A total of 369 patients undergoing MHD treatment for more than 6 months were recruited to participate in this study from the Blood Purification center of our hospital. In this study, MHD patients (CKD Stage 5, end-stage renal failure) were defined as individuals who have been undergoing regular hemodialysis treatment for at least 3 months, with a frequency of 3 times per week (15, 16). The inclusion criteria were as follows: (1) age > 18 years; (2) receiving MHD for 4 h each time, three times per week for at least 3 months; (3) clinical stability (namely, no hospitalization needed within 3 months). The exclusion criteria were as follows: (1) inadequate or irregular dialysis; (2) communication barriers or unwillingness to complete the questionnaire; (3) severe cognitive dysfunction or major psychiatric disorders that may hinder accurate self-reporting or participation in the study; (4) physical disability or illness within 3 months. The study protocol was approved by the Ethics Committee of Wannan Medical College Affiliated Yijishan Hospital (Approved number. 20250214). According to the Declaration of Helsinki, all the participants were informed and signed informed consent prior to the enrollment in this study.

### 2.2 Data collection

A face-to-face interview was conducted to collect demographic information (such as age and gender) as well as clinical characteristics (such as medication use, presence of diabetes and hypertension, and dialysis duration). Clinical data were collected from all the participants during the study at a single time point before the dialysis session. Serum samples were analyzed to measure a range of biochemical parameters, including hemoglobin, 25-hydroxyvitamin D (25(OH)D) level, serum albumin concentration, markers of renal function (such as blood urea nitrogen, serum creatinine, eGFR, and uric acid), lipid profile components [total cholesterol, triglycerides, and low-density lipoprotein cholesterol (LDL-C)], as well as electrolyte levels (sodium, potassium, calcium, magnesium, and phosphorus). C-reactive protein, intact parathyroid hormone (iPTH) levels, and single-pool Kt/V (spKt/V) were also assessed. Two clinical staff members independently verified the accuracy of the data extracted from the electronic medical records. All the patients underwent routine hemodialysis treatments three times per week, with each session lasting 4 h. The dialysis was performed using a “Gambro” hemodialysis machine paired with “Gambro” Polyflux L capillary dialyzers. Access to the vascular system for hemodialysis was primarily achieved through an autologous arteriovenous fistula, which was utilized by 95.4% of the patients. Additionally, 2.4% of patients received dialysis via long-term central venous catheterization, and 2.2% used grafted vascular access. Bicarbonate dialysate was employed during all dialysis sessions.

### 2.3 Depression assessment

The Patient Health Questionnaire (PHQ-9) is a widely used and validated screening tool for depression, applicable across various chronic disease populations, including those with renal disease. Specifically, in patients with ESRD, studies have demonstrated that the PHQ-9 effectively assesses depressive symptoms, showing strong concordance with clinical diagnoses of depression (17–19). Additionally, the PHQ-9 has been shown to correlate well with other depression assessment tools, such as the Beck Depression Inventory (BDI) (19). In this study, the PHQ-9 was used to screen depression in patients with hemodialysis. The PHQ-9 consists of nine self-report items, including (1) loss of interest or pleasure, (2) feelings of sadness or hopelessness, (3) disturbances in sleep, (4) fatigue or lack of energy, (5) changes in appetite, (6) low self-worth, (7) difficulties with concentration, (8) motor restlessness or slowing, and (9) thoughts of self-harm or suicide. This well-established instrument aligns with the nine diagnostic criteria for depression outlined in the Diagnostic and Statistical Manual of Mental Disorders, Fourth Edition (DSM-IV) (17). Each of the items can be scored using a 4-point scale (0 = not at all, 1 = several days, 2 = more than half the days, and 3 = nearly every day). The total score of the nine items ranges from 0 to 27, representing the cumulative frequency of depressive symptoms over the previous 2 weeks. A threshold score of ≥10 has been shown to be both valid and reliable for diagnosing depressive disorder in patients undergoing dialysis, as demonstrated in previous research (18, 19). Additionally, to further explore the relationship between depressive symptom clusters and PhA, we computed subscale scores for the PHQ-9: the cognitive-affective subscale score was derived by summing items 1, 2, 6, 7, and 9, and the somatic subscale score was obtained by summing items 3, 4, 5, and 8 (20).

### 2.4 BIA assessment

Body composition was simultaneously assessed using multifrequency BIA (InBody 770; InBody Co., Ltd., Korea) after the PHQ-9 assessment during the same period (11, 21). BIA measurements were conducted immediately before the patients’ dialysis sessions, ensuring relatively stable fluid conditions to control hydration status and minimize variability. Prior to the BIA measurement, patients were instructed to follow these steps: avoid from eating or drinking for at least 2 h, empty their bladder, remove their socks and shoes, wear clothing of known weight, remove any other items, and relax their whole body. BIA measurements of participants were taken in a static standing position after 5 min of rest. During the assessment, participants were asked to stand barefoot on the metal footplate of the analyzer while gripping the hand electrodes with their arms straight and pointing downward in a neutral position. The hand electrodes were in contact with all five fingers, and the participant’s heels and forefoot were positioned on the circular foot electrode. BIA quantifies body resistance (R) and reactance (Xc) by applying a low-intensity electrical current, with frequencies ranging from 1 to 1,000 kHz, across five distinct body segments: the right arm, left arm, trunk, right leg, and left leg. BIA measurements provided data on various body composition parameters, including phase angle (PhA), skeletal muscle index (SMI), skeletal muscle mass (SMM), body cell mass (BCM), fat free mass (FFM), total body water (TBW), body fat mass (BFM), and body mass index (BMI). The PhA for the entire body was measured at a frequency of 50 kHz. PhA was calculated using the following formula: PhA (°) = arctangent (Xc/R × 180°/*π*), where resistance (R) and reactance (Xc) were the measured values. The SMI was calculated as follows: SMI (kg/m^2^) = SMM (kg)/height^2^ (m^2^).

### 2.5 Statistical analysis

Statistical analyses were performed using IBM SPSS Statistical software for Windows version 27.0 (IBM Corp., Armonk, NY, United States). For quantitative data that satisfied the assumptions of normal distribution and homogeneity of variance, results were presented as mean ± standard deviation (*x̄* ± SD). The independent samples *t*-test was applied for comparisons between two groups, while one-way ANOVA was used for comparisons across multiple groups. For data with non-normal distribution, values were expressed as median (*P_25_*, *P_75_*), with the Mann–Whitney *U* test used for pairwise comparisons and the Kruskal-Wallis *H* test employed for comparisons among multiple groups. Categorical variables (count data) were summarized as frequency and percentage, with the Chi-square test or Fisher’s exact test applied for group comparisons. The Spearman rank correlation test was used to examine associations between the PHQ-9 scores and its sub-scores with various demographic and biochemical factors. Based on the quartiles of PhA values, patients were categorized into four groups: Q1 (2.3° ≤ PhA ≤ 4.1°), Q2 (4.1° < PhA ≤ 4.7°), Q3 (4.7° < PhA ≤ 5.4°), and Q4 (5.4° < PhA ≤ 7.4°). Univariate logistic regression analysis was conducted to identify the potential factors associated with depressive symptoms in MHD patients. Subsequently, multivariate logistic regression models were applied to assess the relationship between PhA values and the presence of depression. A *p*-value of less than 0.05 was considered statistically significant.

## 3 Results

**Figure 1:**
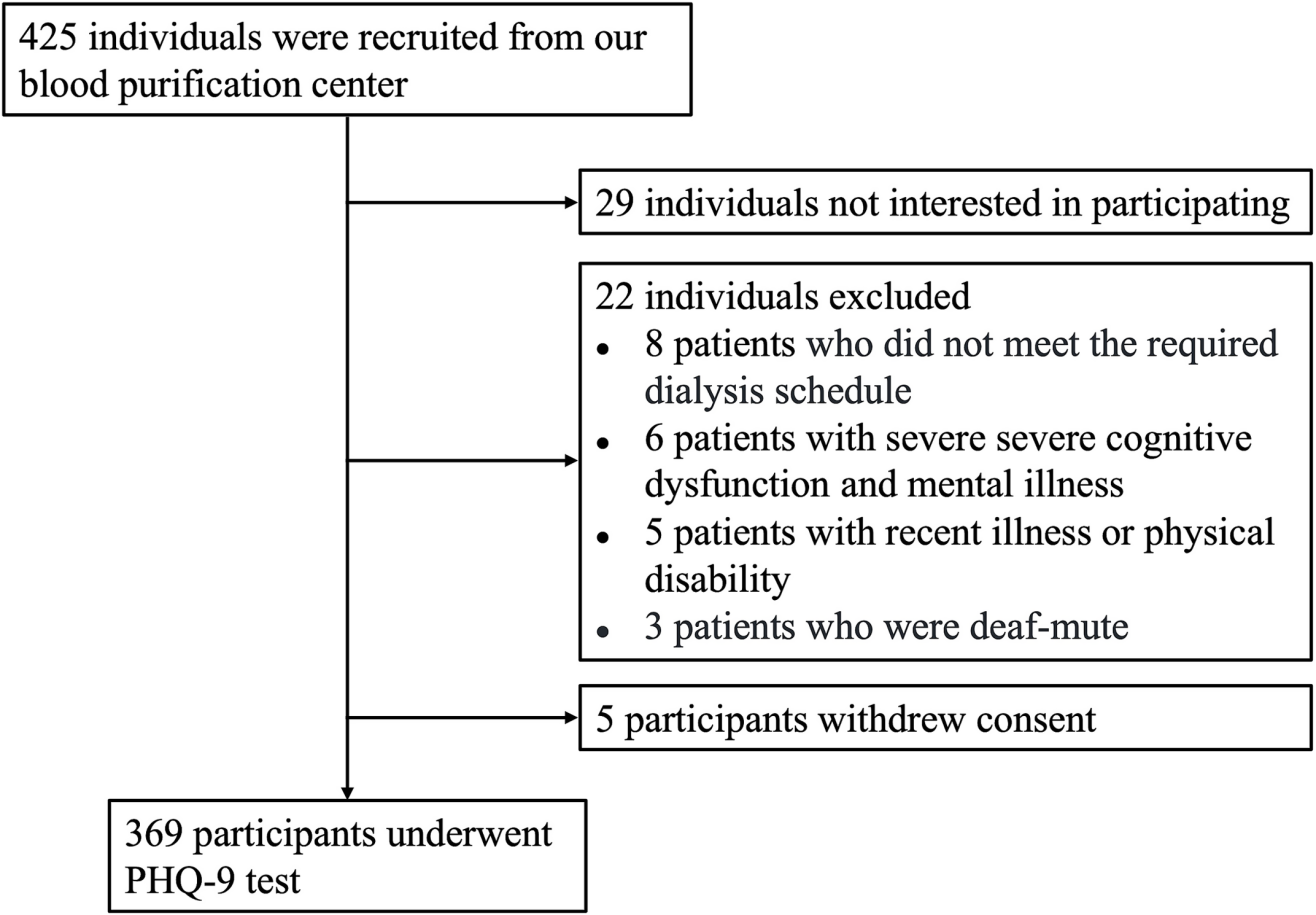
Flow diagram of participant screening. A total of 425 individuals were evaluated for eligibility. Among them, 29 individuals declined to participate, 22 were excluded based on the entry criteria, and 5 withdrew their consent. As a result, 369 participants completed the PHQ-9 testing.

A total of 425 MHD patients were assessed for eligibility to join the study (Figure 1). Among them, 22 individuals were excluded for not meeting the inclusion criteria, which included failure to follow the prescribed dialysis schedule (*n* = 8), severe cognitive dysfunction or mental illness (*n* = 6), recent illness or physical disability (*n* = 5), and being deaf-mute (*n* = 3). In addition, 29 individuals met the criteria but chose not to participate. Consequently, 374 participants initially agreed to take part in the study. However, five participants withdrew their consent before undergoing cognitive testing, leaving 369 participants who completed the full PHQ-9 test.

The demographic, clinical, and body composition characteristics of the study participants were summarized in Table 1, including a comparison between non-depressed and depressed groups of patients receiving MHD. A total of 369 patients were included, with 306 classified as non-depressed and 63 as depressed based on their PHQ-9 scores. The mean age of the participants was 57.52 (median) years, with the depressed group being slightly older (58.50 years) than the non-depressed group (57.28 years), though this difference was not statistically significant (*p* = 0.057). The gender distribution showed a higher proportion of males in the non-depressed group (59.80%) compared to the depressed group (47.60%), but this difference was not statistically significant (*p* = 0.093). Significant differences were observed in medical antecedents between the two groups. A higher percentage of patients in the depressed group had hypertension (79.40% vs. 20.90%), diabetes (31.70% vs. 5.90%), and dyslipidemia (17.50% vs. 2.60%) compared to the non-depressed group (*p* < 0.001 for all). The mean PHQ-9 score was significantly higher in the depressed group (13.55) compared to the non-depressed group (4.20) (*p* < 0.001), confirming the presence of depressive symptoms in this subgroup. In terms of clinical laboratory characteristics, no significant differences between the groups were observed for hemoglobin, creatinine, blood urea nitrogen, uric acid, or electrolyte levels. However, the depressed group had a lower albumin level (40.90 g/L vs. 41.85 g/L, *p* = 0.032). Additionally, the iPTH level was significantly lower in the depressed group (165.50 pg./mL vs. 204.95 pg./mL, *p* = 0.042). Regarding body composition, significant differences were found between the groups. The depressed group had a significantly lower PhA (4.30° vs. 4.89°, *p* < 0.001), SMI (6.17 kg/m^2^ vs. 6.60 kg/m^2^, *p* = 0.005), FFM (41.40 kg vs. 43.65 kg, *p* = 0.040), and TBW (30.30 L vs. 32.13 L, *p* = 0.041). The ECW/TBW ratio was significantly higher in the depressed group (0.39 vs. 0.38, *p* < 0.001), indicating an imbalance in fluid distribution.

**Table 1:** Demographic information, clinical laboratory data, and PHQ-9 score of MHD patients.

Characteristics	Total, *n* = 369	Non-depressed, *n* = 306	Depressed, *n* = 63	*p*-value
Age, years, (range)	57.52 (49.46, 62.90)	57.28 (47.86, 62.83)	58.50 (55.50, 63.90)	0.057
Males, *N* (%)	213 (57.70%)	183 (59.80%)	30 (47.60%)	0.093
Females, *N* (%)	156 (42.30%)	123 (40.20%)	33 (52.40%)	
Antecedents, *N* (%)				
Hypertension	114 (30.90%)	64 (20.90%)	50 (79.40%)	<0.001
Diabetes	38 (10.30%)	18 (5.90%)	20 (31.70%)	<0.001
Dyslipidemia	19 (5.10%)	8 (2.60%)	11 (17.50%)	<0.001
Duration of hemodialysis (months)	84.00 (44.42, 136.36)	84.33 (46.00, 136.50)	79.00 (38.10, 134.83)	0.476
PHQ-9 score	4.80 (2.96, 7.63)	4.20 (2.52, 5.88)	13.55 (11.98, 15.50)	<0.001
Cognitive-affective score	2.53 (1.37, 3.99)	2.14 (1.17, 3.14)	6.96 (5.55, 8.18)	<0.001
Somatic score	2.45 (1.04, 4.38)	1.98 (0.78, 3.19)	6.74 (5.69, 8.11)	<0.001
Depressive symptoms, *N* (%)	63 (17.10%)	0 (0%)	100 (100%)	
Clinical laboratory characteristics				
Hemoglobin, g/L	113.08 (102.89, 123.05)	113.29 (102.85, 123.30)	111.75 (103.25, 122.38)	0.776
Creatinine, μmol/L	793.40 (472.68, 1062.90)	802.45 (476.30, 1070.20)	770.70 (427.53, 974.08)	0.154
eGFR, mL/min/1.73 m^2^	3.63 ± 0.04	3.89 ± 0.06	3.52 ± 0.09	0.127
Blood urea nitrogen, mmol/L	19.60 (10.96, 25.97)	19.91 (10.90, 25.80)	18.87 (11.55, 27.13)	0.888
Uric acid, μmol/L	408.65 ± 123.04	410.09 ± 126.41	401.64 ± 105.74	0.620
Potassium, mmol/L	4.73 (4.04, 5.27)	4.72 (4.01, 5.26)	4.81 (4.19, 5.65)	0.314
Sodium, mmol/L	138.03 ± 2.91	138.11 ± 2.84	137.60 ± 3.22	0.200
Phosphorus, mmol/L	1.78 (1.42, 2.18)	1.81 (1.43, 2.23)	1.64 (1.39, 2.07)	0.201
Calcium, mmol/L	2.28 (2.15, 2.44)	2.28 (2.15, 2.43)	2.34 (2.14, 2.47)	0.555
Magnesium, mmol/L	1.04 (0.96, 1.13)	1.04 (0.96, 1.12)	1.04 (0.94, 1.13)	0.630
Albumin, g/L	41.66 (39.12, 43.56)	41.85 (39.28, 43.69)	40.90 (38.33, 42.79)	0.032
β2-microglobulin, mg/L	35.62 (29.89, 44.82)	35.93 (30.07, 45.10)	34.80 (28.28, 42.73)	0.548
Iron, μmol/L	12.87 (9.34, 17.69)	13.23 (9.60, 18.10)	12.00 (7.83, 16.98)	0.070
Ferritin, μg/L	275.00 (59.60, 1635.00)	272.50 (67.10, 1740.00)	283.00 (50.95, 1417.50)	0.852
Transferrin, g/L	1.53 (1.22, 1.93)	1.54 (1.22, 1.92)	1.53 (1.23, 1.99)	0.868
C-Reactive protein, mg/L	3.18 (1.35, 7.18)	3.03 (1.30, 6.84)	3.86 (2.00, 7.91)	0.139
iPTH, pg./mL	199.70 (107.28, 377.25)	204.95 (115.10, 387.10)	165.50 (65.20, 317.75)	0.042
Triglyceride, mmol/L	1.47 (1.07, 2.22)	1.45 (1.08, 2.21)	1.54 (1.05, 2.43)	0.995
Total cholesterol, mmol/L	3.96 ± 1.09	3.97 ± 1.07	3.91 ± 1.17	0.704
LDL-C, mmol/L	2.33 (1.77, 2.80)	2.36 (1.80, 2.77)	2.21 (1.63, 2.97)	0.512
25(OH)D, ng/mL	51.02 (38.51, 66.43)	51.45 (38.93, 66.34)	49.90 (36.73, 66.31)	0.691
spKt/V	1.24 (1.19, 1.40)	1.24 (1.20, 1.40)	1.22 (1.18, 1.35)	0.299
Body composition				
PhA, degree, ^o^	4.79 ± 0.97	4.89 ± 0.94	4.30 ± 0.98	<0.001
BMI, kg/m^2^	22.31 (19.83, 24.49)	22.32 (19.80, 24.64)	22.30 (19.91, 23.98)	0.416
SMI, kg/m^2^	6.53 ± 1.11	6.60 ± 1.10	6.17 ± 1.09	0.005
FFM, kg	43.20 (37.87, 49.93)	43.65 (38.10, 50.40)	41.40 (36.05, 46.77)	0.040
TBW, L	31.92 (27.84, 36.91)	32.13 (28.00, 37.17)	30.30 (26.45, 34.58)	0.041
ECW/TBW	0.39 (0.37, 0.39)	0.38 (0.37, 0.39)	0.39 (0.38, 0.40)	<0.001
BFM, kg	16.50 (11.09, 22.12)	16.13 (10.94, 22.21)	17.65 (12.43, 20.72)	0.568

Quantitative data with a normal distribution and homogeneity of variance are presented as mean ± standard deviation (*x̄* ± SD). For data that do not follow a normal distribution, values are expressed as median (*P*_25_, *P*_75_). Categorical variables (count data) are presented as frequencies and percentages.

25(OH)D, 25-hydroxyvitamin D; LDL-C, low-density lipoprotein cholesterol; iPTH, intact parathyroid hormone; spKt/V, single-pool Kt/V; PhA, phase angle; BMI, body mass index; SMI, skeletal muscle index; FFM, fat free mass; TBW, total body water; ECW/TBW, extracellular water/total body water; BFM, body fat mass.

**Figure 2:**
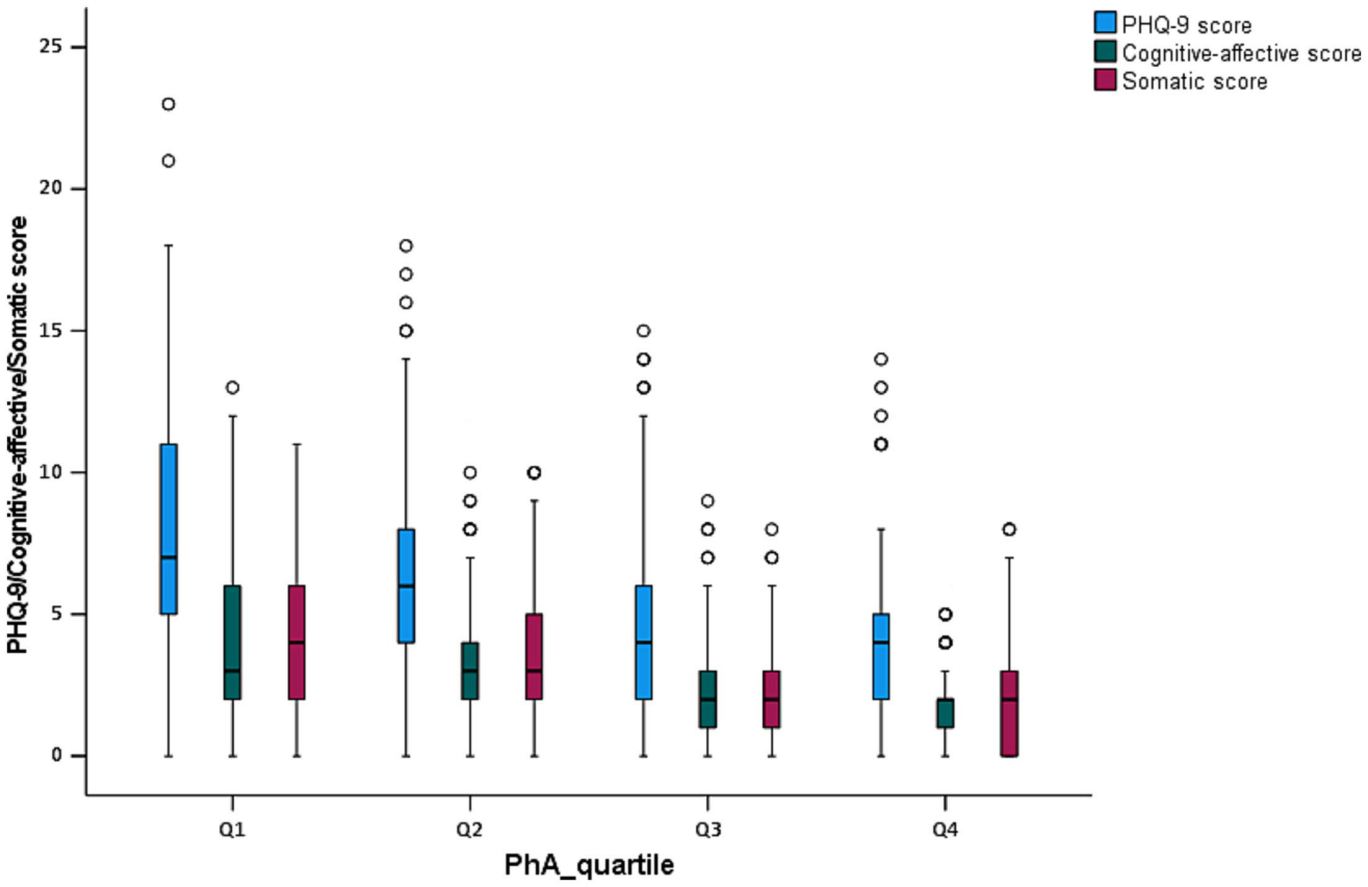
Box plot analysis of PHQ-9 score, cognitive-affective score, and somatic score by PhA quartiles in MHD patients.

The clinical, biochemical, and body composition characteristics of patients across the four groups (Q1–Q4) categorized by quartiles of PhA values were presented in Table 2. Significant differences were observed in several variables among the groups. In terms of demographics, the age of participants significantly decreased across the groups (Q1: 61.00 years, Q4: 53.83 years, *p* < 0.001). The proportion of males increased notably from 49.50% in Q1 to 77.20% in Q4 (*p* < 0.001), whereas the proportion of females decreased from 50.50% in Q1 to 22.80% in Q4 (*p* < 0.001). The prevalence of clinical comorbidities, including hypertension, diabetes, and dyslipidemia, did not show significant differences between the groups (*p* > 0.05). The duration of hemodialysis was similar across the quartiles (*p* = 0.535). Regarding depressive symptoms, the PHQ-9 scores were highest in Q1 (6.94), and progressively decreased through Q4 (3.46), with a significant trend observed (*p* < 0.001). Depressive symptoms, defined as a PHQ-9 score ≥ 10, were reported in 30.90% of patients in Q1, 20.00% in Q2, 10.00% in Q3, and 6.50% in Q4, again showing a significant reduction (*p* < 0.001). Part of biochemical parameters demonstrated significant differences across the PhA quartiles. Creatinine levels were the lowest in Q1 (663.00 μmol/L) and highest in Q4 (1041.70 μmol/L), showing a significant increase from Q1 to Q4 (*p* < 0.001). Blood urea nitrogen levels also rose progressively from Q1 to Q4 (*p* = 0.015), while uric acid levels exhibited a gradual increase from 381.51 μmol/L in Q1 to 433.89 μmol/L in Q4 (*p* = 0.008). Phosphorus levels were significantly higher in the lower PhA quartiles, with a notable difference observed across groups (*p* < 0.001). Additionally, albumin, triglyceride, and magnesium levels showed significant variation between quartiles, with higher PhA quartiles associated with elevated albumin levels (*p* < 0.001) and a trend toward increased triglyceride and magnesium levels (*p* = 0.001 and 0.026, respectively). Conversely, other laboratory parameters, such as hemoglobin, potassium, sodium, calcium, ferritin, iPTH, and spKt/V, did not show significant differences across the quartiles (*p* > 0.05). In terms of body composition, PhA significantly increased across the groups with values rising from 3.60° in Q1 to 6.07° in Q4 (*p* < 0.001). BMI increased progressively from 20.60 in Q1 to 23.73 in Q4 (*p* < 0.001), and SMI also gradually increased from 6.13 in Q1 to 7.21 in Q4 (*p* < 0.001). FFM, TBW, and BFM did not exhibit a continuously increasing pattern but showed significant differences between the groups (*p* < 0.05). Conversely, ECW/TBW showed a decreased trend with significant differences across the groups (*p* < 0.001). Additionally, compared to patients in the Q3 and Q4 groups, patients in the lower quartile groups of PhA (Q1 and Q2) presented the significantly higher cognitive-affective scores and somatic scores with *p* < 0.001 (Figure 2 and Table 2).

**Table 2:** Clinical, biochemical characteristics, and PHQ-9 scores of different groups.

Variable	Q1 (*n* = 97)	Q2 (*n* = 90)	Q3 (*n* = 90)	Q4 (*n* = 92)	*p* value
Age, years, (range)	61.00 (54.72, 67.94)	59.83 (53.67, 67.50)	56.64 (47.50, 62.27)	53.83 (45.25, 58.71)	<0.001
Males, *N* (%)	48 (49.50%)	45 (50.00%)	49 (54.40%)	71 (77.20%)	<0.001
Females, *N* (%)	49 (50.50%)	45 (50.00%)	41 (45.60%)	21 (22.80%)	<0.001
Antecedents, *N* (%)					
Hypertension	30 (30.90%)	29 (32.20%)	27 (30.00%)	28 (30.40%)	0.992
Diabetes	13 (13.40%)	9 (10.00%)	7 (7.80%)	9 (9.80%)	0.650
Dyslipidemia	7 (7.20%)	3 (3.30%)	5 (5.60%)	4 (4.30%)	0.674
Duration of hemodialysis (months)	86.00 (45.75, 149.50)	87.75 (43.00, 138.00)	85.50 (50.00, 131.00)	70.50 (39.67, 129.50)	0.535
PHQ-9 score	6.94 (4.60, 11.35)	5.61 (3.68, 8.40)	4.08 (2.23, 6.13)	3.46 (2.03, 4.84)	<0.001
Cognitive-affective score	3.58 (2.21, 5.75)	2.97 (1.66, 4.55)	2.14 (1.17, 3.28)	1.80 (0.97, 2.73)	<0.001
Somatic score	3.63 (2.05, 5.67)	2.74 (1.42, 4.86)	1.88 (0.59, 3.67)	1.67 (0.48, 2.94)	<0.001
Depressive symptoms, *N* (%)	30 (30.90%)	18 (20.00%)	9 (10.00%)	6 (6.50%)	<0.001
Clinical laboratory characteristics					
Hemoglobin, g/L	113.80 (101.63, 123.63)	110.63 (101.80, 121.40)	113.55 (104.67, 123.40)	114.38 (103.50, 124.80)	0.551
Creatinine, μmol/L	663.00 (404.18, 905.80)	733.70 (378.70, 963.20)	822.90 (508.30, 1110.80)	1041.70 (616.95, 1232.80)	<0.001
eGFR, mL/min/1.73 m^2^	3.12 ± 0.05	3.35 ± 0.11	3.59 ± 0.08	3.77 ± 0.04	<0.001
Blood urea nitrogen, mmol/L	17.85 (9.23, 24.26)	17.42 (8.70, 24.84)	20.13 (10.80, 26.54)	22.70 (14.37, 27.81)	0.015
Uric acid, μmol/L	381.51 ± 109.29	394.44 ± 122.69	426.31 ± 125.41	433.89 ± 128.74	0.008
Potassium, mmol/L	4.83 (4.22, 5.49)	4.52 (3.78, 5.09)	4.61 (3.99, 5.37)	4.94 (4.14, 5.19)	0.089
Sodium, mmol/L	137.56 ± 3.23	137.89 ± 2.91	138.14 ± 2.80	138.54 ± 2.57	0.125
Phosphorus, mmol/L	1.76 (1.37, 2.15)	1.63 (1.28, 1.95)	1.85 (1.54, 2.33)	1.96 (1.52, 2.30)	<0.001
Calcium, mmol/L	2.28 (2.15, 2.43)	2.29 (2.13, 2.42)	2.26 (2.15, 2.43)	2.32 (2.17, 2.46)	0.558
Magnesium, mmol/L	1.00 (0.93, 1.09)	1.05 (0.96, 1.15)	1.06 (1.00, 1.14)	1.03 (0.96, 1.12)	0.026
Albumin, g/L	39.57 (37.38, 41.76)	40.90 (38.80, 43.23)	42.55 (41.13, 44.30)	42.63 (40.70, 44.07)	<0.001
β2-Microglobulin, mg/L	35.50 (31.38, 44.40)	34.95 (29.50, 45.40)	36.60 (29.70, 42.80)	35.70 (29.70, 45.10)	0.961
Iron, μmol/L	12.20 (8.75, 17.68)	13.55 (9.13, 17.60)	12.73 (9.90, 17.47)	13.20 (9.45, 18.40)	0.722
Ferritin, μg/L	444.00 (58.05, 2645.00)	303.00 (54.60, 2010.00)	303.00 (70.60, 1210.00)	219.50 (58.50, 945.50)	0.391
Transferrin, g/L	1.47 (1.18, 1.93)	1.53 (1.19, 1.87)	1.46 (1.19, 1.88)	1.64 (1.29, 2.06)	0.157
C-Reactive protein, mg/L	3.93 (1.78, 7.83)	3.11 (1.37, 7.50)	2.65 (1.11, 5.80)	2.97 (1.29, 7.00)	0.163
iPTH, pg./mL	185.10 (101.45, 354.70)	170.40 (83.10, 338.20)	201.60 (108.60, 351.10)	243.50 (138.95, 504.95)	0.051
Triglyceride, mmol/L	1.18 (0.92, 1.71)	1.55 (1.17, 2.22)	1.47 (1.14, 2.40)	1.74 (1.12, 2.64)	0.001
Total cholesterol, mmol/L	3.77 ± 1.11	3.95 ± 1.22	4.08 ± 0.96	4.06 ± 1.04	0.178
LDL-C, mmol/L	2.14 (1.57, 2.86)	2.28 (1.68, 2.80)	2.37 (1.93, 2.65)	2.52 (1.94, 2.86)	0.070
25(OH)D, ng/mL	48.91 (39.13, 64.39)	48.20 (36.24, 62.35)	51.64 (37.70, 67.44)	54.58 (41.66, 72.02)	0.204
spKt/V	1.24 (1.19, 1.43)	1.23 (1.19, 1.40)	1.27 (1.17, 1.40)	1.22 (1.19, 1.36)	0.850
Body composition					
PhA, degree, ^o^	3.60 ± 0.42	4.47 ± 0.17	5.07 ± 0.20	6.07 ± 0.49	<0.001
BMI, kg/m^2^	20.60 (18.18, 23.05)	21.63 (19.84, 24.05)	22.87 (19.47, 24.50)	23.73 (21.60, 26.10)	<0.001
SMI, kg/m^2^	6.13 ± 1.33	6.31 ± 0.96	6.48 ± 0.92	7.21 ± 0.86	<0.001
FFM, kg	41.00 (34.70, 45.30)	40.25 (36.33, 48.40)	42.25 (38.00, 47.60)	48.00 (42.60, 54.55)	<0.001
TBW, L	30.20 (25.45, 33.53)	29.70 (26.73, 35.93)	31.05 (28.00, 35.10)	35.20 (31.20, 40.05)	<0.001
ECW/TBW	0.40 (0.39, 0.41)	0.39 (0.38, 0.40)	0.38 (0.37, 0.39)	0.37 (0.36, 0.38)	<0.001
BFM, kg	13.80 (9.48, 19.15)	16.67 (10.80, 23.30)	18.10 (12.70, 23.00)	17.27 (12.33, 21.75)	0.002

Quantitative data with a normal distribution and homogeneity of variance are presented as mean ± standard deviation (*x̄* ± SD). For data that do not follow a normal distribution, values are expressed as median (*P*_25_, *P*_75_). Categorical variables (count data) are presented as frequencies and percentages.

25(OH)D, 25-hydroxyvitamin D; LDL-C, low-density lipoprotein cholesterol; iPTH, intact parathyroid hormone; spKt/V, single-pool Kt/V; PhA, phase angle; BMI, body mass index; SMI, skeletal muscle index; FFM, fat free mass; TBW, total body water; ECW/TBW, extracellular water/total body water; BFM, body fat mass.

The Spearman rank correlation analysis between PHQ-9 scores and various clinical and biochemical characteristics, as well as body composition parameters, was summarized in Table 3. The results indicated a significant positive correlation between PHQ-9 scores and age (*ρ* = 0.454, *p* < 0.001), suggesting that older patients tend to have higher depressive symptoms. Moreover, significant correlations were observed between PHQ-9 scores and the presence of hypertension (*ρ* = 0.315, *p* < 0.001), diabetes (*ρ* = 0.173, *p* < 0.001), and dyslipidemia (*ρ* = 0.158, *p* = 0.002), all of which were common comorbidities in patients undergoing hemodialysis. The analysis also revealed a negative correlation between PHQ-9 scores and phase angle (PhA) values (*ρ* = −0.394, *p* < 0.001), indicating that higher PhA values were associated with lower levels of depressive symptoms. This correlation suggests that better nutritional status and cellular integrity, which are reflected by higher PhA, might be linked to improved mental health in these patients. Similarly, significant negative correlations were found with SMI (*ρ* = −0.158, *p* = 0.002), FFM (*ρ* = −0.136, *p* = 0.009), TBW (*ρ* = −0.132, *p* = 0.011), and ECW/TBW (*ρ* = 0.366, *p* < 0.001). These findings suggest that patients with better body composition, including higher muscle mass and a more favorable fluid balance, tend to exhibit fewer depressive symptoms. In terms of biochemical parameters, the PHQ-9 score showed a negative correlation with creatinine (*ρ* = −0.154, *p* = 0.003), but no significant correlation with blood urea nitrogen, uric acid, or other biochemical parameter levels. Additionally, albumin levels were negatively correlated with PHQ-9 scores (*ρ* = −0.128, *p* = 0.014), further supporting the association between better nutritional status and reduced depressive symptoms.

**Table 3:** Correlation analysis between PHQ-9 score and others parameters using Spearman rank correlation analysis.

Characteristics	PHQ-9 score	
	*ρ*	*P*
Age (years)	0.454	<0.001
Sex, *N* (%)	−0.089	0.089
Antecedents, *N* (%)		
Hypertension	0.315	<0.001
Diabetes	0.173	<0.001
Dyslipidemia	0.158	0.002
Duration of hemodialysis (months)	−0.097	0.064
Clinical laboratory characteristics		
Hemoglobin, g/L	0.020	0.699
Creatinine, μmol/L	−0.154	0.003
Blood urea nitrogen, mmol/L	−0.031	0.551
Uric acid, μmol/L	−0.076	0.143
Potassium, mmol/L	0.007	0.896
Sodium, mmol/L	−0.072	0.167
Phosphorus, mmol/L	−0.040	0.445
Calcium, mmol/L	0.073	0.161
Magnesium, mmol/L	0.039	0.453
Albumin, g/L	−0.128	0.014
β2-microglobulin, mg/L	−0.050	0.336
Iron, μmol/L	−0.087	0.094
Ferritin, μg/L	−0.067	0.200
Transferrin, g/L	0.082	0.115
C-Reactive protein, mg/L	0.083	0.111
iPTH, pg./mL	−0.081	0.121
Triglyceride, mmol/L	−0.043	0.410
Total cholesterol, mmol/L	−0.038	0.467
LDL-C, mmol/L	−0.060	0.254
25(OH)D, ng/mL	0.014	0.789
spKt/V	−0.056	0.286
Body composition		
PhA, degree, ^o^	−0.394	<0.001
BMI, kg/m^2^	−0.077	0.139
SMI, kg/m^2^	−0.158	0.002
FFM, kg	−0.136	0.009
TBW, L	−0.132	0.011
ECW/TBW	0.366	<0.001
BFM, kg	−0.004	0.983

25(OH)D, 25-hydroxyvitamin D; LDL-C, low-density lipoprotein cholesterol; iPTH, intact parathyroid hormone; spKt/V, single-pool Kt/V; PhA, phase angle; BMI, body mass index; SMI, skeletal muscle index; FFM, fat free mass; TBW, total body water; ECW/TBW, extracellular water/total body water; BFM, body fat mass.

Furthermore, the association between clinical, biochemical, body composition parameters, and depressive symptoms in MHD patients was analyzed using the binary logistic regression analysis (Table 4). All the variables with *p* < 0.05 in univariate models, including hypotension, diabetes, dyslipidemia, albumin, PhA, SMI, FFM, TBW, and ECW/TBW, were considered and included when conducting the multivariate regression analysis. The univariate analysis showed a strong association between PhA and depressive symptoms with an odds ratio (OR) of 0.505 (95% CI: 0.368–0.692, *p* < 0.001). Multivariate analysis confirmed this relationship with yielding an OR of 0.072 (95% CI: 0.015–0.339, *p* < 0.001), suggesting that higher PhA values were inversely correlated with depressive symptoms in MHD patients. Among the clinical parameters, hypertension (OR: 22.264, 95% CI: 9.436–53.475, *p* < 0.001) and diabetes (OR: 5.200, 95% CI: 1.963–13.774, *p* < 0.001) were significantly associated with depressive symptoms in both univariate and multivariate analyses. Other clinical parameters such as age, gender, and duration of hemodialysis were not significantly associated with depressive symptoms in the multivariate model. Biochemical parameters such as albumin (OR: 0.930, 95% CI: 0.869–0.994, *p* = 0.034) were marginally associated with depressive symptoms in univariate analysis but did not reach statistical significance in the multivariate model (OR: 0.919, 95% CI: 0.831–1.016, *p* = 0.100). Others biochemical parameters, such as hemoglobin, lipid profile components, electrolyte levels, markers of renal function, C-reactive protein, and iPTH, showed no significant relationship (*p* > 0.05) with depression in MHD patients. Additionally, body composition parameters showed notable correlations with depressive symptoms. PhA and body composition indicators such as SMI, FFM, and TBW were significantly associated with depressive symptoms. Specifically, the ECW/TBW ratio was positively correlated with depressive symptoms (OR: 1.873, 95% CI: 1.024–3.391, *p* = 0.002), but the association weakened after adjusting for other variables in the multivariate analysis (OR: 0.003, 95% CI: 0.001–0.181, *p* = 0.046).

**Table 4:** Correlation analysis between clinical, biochemical parameters and depression in MHD patients through logistic regression analysis.

Variable	Univariate analysis		Multivariate analysis	
	OR (95% *CI*)	*P*-value	OR (95% *CI*)	*P*-value
Age (years)	1.021 (0.998–1.045)	0.071		
Females, *N* (%)	Ref.			
Males, *N* (%)	0.611 (0.354–1.053)	0.076		
Antecedents, *N* (%)				
Hypertension	14.543 (7.446–28.407)	<0.001	22.264 (9.436–53.475)	<0.001
Diabetes	7.442 (3.648–15.180)	<0.001	5.200 (1.963–13.774)	<0.001
Dyslipidemia	7.880 (3.026–20.521)	<0.001	3.509 (0.983–12.527)	0.053
Duration of hemodialysis (months)	0.999 (0.994–1.003)	0.539		
Clinical laboratory characteristics				
Hemoglobin, g/L	0.997 (0.981–1.013)	0.738		
Creatinine, μmol/L	0.999 (0.999–1.000)	0.164		
Blood urea nitrogen, mmol/L	1.004 (0.976–1.033)	0.774		
Uric acid, μmol/L	0.999 (0.997–1.002)	0.619		
Potassium, mmol/L	1.179 (0.891–1.561)	0.248		
Sodium, mmol/L	0.941 (0.857–1.033)	0.201		
Phosphorus, mmol/L	0.734 (0.446–1.209)	0.225		
Calcium, mmol/L	1.060 (0.372–3.021)	0.913		
Magnesium, mmol/L	0.357 (0.044–2.904)	0.336		
Albumin, g/L	0.930 (0.869–0.994)	0.034	0.919 (0.831–1.016)	0.100
β2-microglobulin, mg/L	0.996 (0.975–1.018)	0.726		
Iron, μmol/L	0.971 (0.933–1.010)	0.145		
Ferritin, μg/L	1.000 (1.000–1.000)	0.404		
Transferrin, g/L	1.040 (0.658–1.642)	0.867		
C-Reactive protein, mg/L	1.008 (0.993–1.023)	0.281		
iPTH, pg./mL	0.999 (0.998–1.000)	0.157		
Triglyceride, mmol/L	0.966 (0.779–1.199)	0.755		
Total cholesterol, mmol/L	0.952 (0.741–1.224)	0.703		
LDL-C, mmol/L	0.904 (0.635–1.2870)	0.575		
25(OH)D, ng/mL	0.998 (0.987–1.009)	0.664		
spKt/V	0.596 (0.270–1.318)	0.201		
Body composition				
PhA, degree, ^o^	0.505 (0.368–0.692)	<0.001	0.072 (0.015–0.339)	<0.001
BMI, kg/m^2^	0.959 (0.891–1.033)	0.272		
SMI, kg/m^2^	0.690 (0.532–0.895)	0.005	4.068 (0.800–20.686)	0.091
FFM, kg	0.962 (0.931–0.995)	0.025	13.126 (0.800–215.433)	0.071
TBW, L	0.950 (0.908–0.993)	0.025	0.026 (0.001–1.263)	0.065
ECW/TBW	1.873 (1.024–3.391)	0.002	0.003 (0.001–0.181)	0.046
BFM, kg	1.007 (0.972–1.042)	0.711		

25(OH)D, 25-hydroxyvitamin D; LDL-C, low-density lipoprotein cholesterol; iPTH, intact parathyroid hormone; spKt/V, single-pool Kt/V; PhA, phase angle; BMI, body mass index; SMI, skeletal muscle index; FFM, fat free mass; TBW, total body water; ECW/TBW, extracellular water/total body water; BFM, body fat mass.

The association between PhA and depressive symptoms in MHD patients was further analyzed across four models using the Q4 group (normal PhA value) as a reference (Table 5). The unadjusted analysis (Model 1) showed a strong association between lower PhA values (Q1 and Q2) and increased odds of depressive symptoms. Specifically, patients in the Q1 group had an OR of 6.418 (95% CI: 2.525–16.313, *p* < 0.001), and those in the Q2 group had an OR of 3.583 (95% CI: 1.351–9.505, *p* < 0.001), indicating a significant risk of depression compared to the Q4 group. In Model 2, which was adjusted for age, sex, and duration of hemodialysis, the association remained significant for Q1 and Q2, with ORs of 6.051 (95% CI: 2.280–16.055, *p* < 0.001) and 3.309 (95% CI: 1.206–9.078, *p* = 0.020), respectively. However, the association weakened in the higher quartiles (Q3) with no significant relationship observed in Model 1 (OR: 1.593, 95% CI: 0.543–4.674, *p* = 0.397) and Model 2 (OR: 1.488, 95% CI: 0.500–4.428, *p* = 0.475). After further adjustment for comorbidities such as hypertension, diabetes, and dyslipidemia (Model 3), the ORs remained statistically significant for Q1 (OR: 12.548, 95% CI: 3.666–42.947, *p* < 0.001) and Q2 (OR: 5.482, 95% CI: 1.644–18.279, *p* = 0.006). The final model (Model 4), which accounted for a comprehensive set of clinical and biochemical parameters, revealed that patients had an OR of 51.760 (95% CI: 4.068–658.592, *p* = 0.002) in the Q1 group and an OR of 14.798 (95% CI: 2.268–96.538, *p* = 0.005) in the Q2 group, further reinforcing the robust association between low PhA and depressive symptoms in MHD patients.

**Table 5:** The association between PhA and depression in MHD patients.

Group	Model 1		Model 2		Model 3		Model 4	
	OR (95% *CI*)	*P*-value	OR (95% *CI*)	*P*-value	OR (95% *CI*)	*P*-value	OR (95% *CI*)	*P*-value
Q1: 2.3°–4.1°	6.418 (2.525–16.313)	<0.001	6.051 (2.280–16.055)	<0.001	12.548 (3.666–42.947)	<0.001	51.760 (4.068–658.592)	0.002
Q2: 4.2°–4.7°	3.583 (1.351–9.505)	0.01	3.309 (1.206–9.078)	0.020	5.482 (1.644–18.279)	0.006	14.798 (2.268–96.538)	0.005
Q3: 4.8°–5.4°	1.593 (0.543–4.674)	0.397	1.488 (0.500–4.428)	0.475	2.066 (0.579–7.379)	0.264	3.960 (0.814–19.267)	0.088
Q4: 5.5°–7.4°	1.00	–	1.00	–	1.00	–	1.00	–

Model 1: Unadjusted model.

Model 2: Adjusted age, sex, and duration of HD based on Model 1.

Model 3: Adjusted hypertension, diabetes, and dyslipidemia based on Model 2.

Model 4: Adjusted albumin, SMI, FFM, TBW, and ECW/TBW based on Model 3.

In addition, the gender-specific analyses of PhA and depressive symptoms in MHD patients were further conducted and presented in Tables 6–8. Table 6 provides the demographic, clinical laboratory, and PHQ-9 score characteristics of the MHD patients stratified by gender. The analysis revealed that there were significant gender differences in the duration of hemodialysis (*p* < 0.001), albumin levels (*p* = 0.009), and ferritin levels (*p* < 0.001), with females exhibiting a higher ferritin concentration. There was no significant difference in depressive symptoms between genders (*p* = 0.089). Notably, the PhA was significantly lower in females compared to males (4.51 ± 0.83° vs. 4.99 ± 1.02°, *p* < 0.001), as well as BMI, SMI, FFM, and TBW. The correlation analysis between PHQ-9 scores and BIA parameters based on gender difference was shown in Table 7. For both males and females, PhA exhibited a significant negative correlation with PHQ-9 scores (*ρ* = −0.446, *p* < 0.001 for females; *ρ* = −0.335, *p* < 0.001 for males), suggesting that lower PhA values were associated with higher depressive symptoms. Additionally, cognitive-affective and somatic scores of depression showed strong positive correlations with PHQ-9 scores for both genders (*ρ* = 0.885, *p* < 0.001 for females; *ρ* = 0.809, *p* < 0.001 for males), further supporting the relationship between depressive symptoms and body composition parameters. Other parameters, such as SMI and ECW/TBW, showed significant correlations with depressive symptoms by gender difference. Table 8 presented the results of the binary logistic regression analysis, which examined the association between PhA and depression. For males, a lower PhA (Q1: 2.3°–4.1°) was strongly associated with depressive symptoms (OR = 4.400, 95% CI: 1.436–13.479, *p* = 0.009), but the association weakened in higher PhA categories and Model 4 with confounder adjustments (*p* = 0.193). Conversely, for females, a stronger association was observed in the lowest PhA category (Q1: 2.3°–4.1°), with an OR of 11.613 (95% CI: 1.435–93.951, *p* = 0.022), which remained significant even after adjusting for confounders from Model 1 to Model 4 (OR = 80.724, 95% CI: 4.488–859.044, *p* = 0.008). These findings suggest that a lower PhA may be a more robust predictor of depressive symptoms in females than in males.

**Table 6:** Demographic information, clinical laboratory data, and PHQ-9 score of MHD patients based on gender difference.

Characteristics	Females, *n* = 156	Males, *n* = 213	*P*-value
Age, years, (range)	58.00 (52.25, 62.00)	57.00 (48.00, 63.00)	0.242
Antecedents, *N* (%)			
Hypertension	48 (30.80%)	66 (31.00%)	0.965
Diabetes	20 (12.80%)	18 (8.50%)	0.173
Dyslipidemia	6 (3.80%)	13 (6.10%)	0.333
Duration of hemodialysis (months)	105.00 (54.00, 153.00)	68.00 (40.00, 112.50)	<0.001
PHQ-9 score	5.00 (3.00, 9.00)	5.00 (3.00, 7.00)	0.089
Cognitive-affective score	3.00 (2.00, 4.00)	2.00 (1.00, 4.00)	0.059
Somatic score	3.00 (1.00, 5.00)	2.00 (1.00, 4.00)	0.105
Depressive symptoms, *N* (%)	33 (21.20%)	30 (14.10%)	
Clinical laboratory characteristics			
Hemoglobin, g/L	112.50 (103.00, 123.00)	113.00 (103.00, 124.00)	0.864
Creatinine, μmol/L	699.10 (321.00, 948.05)	868.60 (561.50, 1146.55)	<0.001
Blood urea nitrogen, mmol/L	18.71 (7.65, 26.71)	20.13 (12.39, 25.74)	0.199
Uric acid, μmol/L	411.72 ± 125.30	406.40 ± 121.60	0.873
Potassium, mmol/L	4.78 (4.00, 5.25)	4.65 (4.10, 5.30)	0.978
Sodium, mmol/L	137.86 ± 2.74	138.15 ± 3.02	0.275
Phosphorus, mmol/L	1.71 (1.38, 2.10)	1.80 (1.44, 2.18)	0.513
Calcium, mmol/L	2.32 (2.16, 2.48)	2.27 (2.14, 2.42)	0.084
Magnesium, mmol/L	1.05 (0.98, 1.14)	1.03 (0.95, 1.11)	0.119
Albumin, g/L	41.20 (38.80, 42.80)	42.10 (39.20, 44.00)	0.009
β2-microglobulin, mg/L	35.50 (29.80, 44.65)	35.70 (30.00, 44.90)	0.577
Iron, μmol/L	13.05 (9.60, 18.83)	12.90 (9.20, 16.55)	0.201
Ferritin, μg/L	614.50 (92.15, 2777.50)	194.00 (54.00, 869.00)	<0.001
Transferrin, g/L	1.46 (1.14, 1.86)	1.59 (1.27, 2.01)	0.009
C-Reactive protein, mg/L	3.14 (1.31, 6.66)	3.25 (1.36, 7.43)	0.529
iPTH, pg./mL	152.50 (66.78, 297.28)	227.50 (139.20, 431.35)	<0.001
Triglyceride, mmol/L	1.64 (1.17, 2.30)	1.36(1.00, 2.15)	0.009
Total cholesterol, mmol/L	4.29 ± 1.08	3.72 ± 1.04	0.994
LDL-C, mmol/L	2.39 (1.93, 3.03)	2.19 (1.62, 2.963)	<0.001
25(OH)D, ng/mL	45.65 (35.71, 61.78)	56.04 (42.17, 69.06)	<0.001
spKt/V	1.26 (1.20, 1.49)	1.22 (1.20, 1.37)	0.026
Body composition			
PhA, degree, ^o^	4.51 ± 0.83	4.99 ± 1.02	<0.001
BMI, kg/m^2^	22.00 (19.33, 23.88)	22.60 (20.10, 25.05)	0.005
SMI, kg/m^2^	5.69 ± 0.73	7.14 ± 0.93	<0.001
FFM, kg	37.50FF (34.03, 39.90)	48.40 (44.20, 53.55)	<0.001
TBW, L	27.50 (24.93, 29.45)	35.80 (32.75, 39.50)	<0.001
ECW/TBW	0.39 (0.38, 0.40)	0.39 (0.38, 0.40)	0.120
BFM, kg	17.80 (12.15, 22.30)	15.60 (10.55, 21.45)	0.090

Quantitative data with a normal distribution and homogeneity of variance are presented as mean ± standard deviation (*x̄* ± SD). For data that do not follow a normal distribution, values are expressed as median (*P*_25_, *P*_75_). Categorical variables (count data) are presented as frequencies and percentages.

25(OH)D, 25-hydroxyvitamin D; LDL-C, low-density lipoprotein cholesterol; iPTH, intact parathyroid hormone; spKt/V, single-pool Kt/V; PhA, phase angle; BMI, body mass index; SMI, skeletal muscle index; FFM, fat free mass; TBW, total body water; ECW/TBW, extracellular water/total body water; BFM, body fat mass.

**Table 7:** The correlation analysis between PHQ-9 score and others parameters based on gender difference using Spearman rank correlation analysis.

Characteristics	PHQ-9 score			
	Females		Males	
	*ρ*	*P*	*ρ*	*P*
PhA, degree, ^o^	−0.446	<0.001	−0.335	<0.001
Cognitive-affective score	0.885	<0.001	0.809	<0.001
Somatic score	0.906	<0.001	0.878	<0.001
BMI, kg/m^2^	−0.090	0.266	−0.057	0.405
SMI, kg/m^2^	−0.187	0.019	−0.121	0.078
FFM, kg	−0.056	0.486	−0.139	0.043
TBW, L	−0.053	0.508	−0.130	0.058
ECW/TBW	0.367	<0.001	0.354	<0.001
BFM, kg	−0.006	0.943	−0.022	0.752

25(OH)D, 25-hydroxyvitamin D; LDL-C, low-density lipoprotein cholesterol; iPTH, intact parathyroid hormone; spKt/V, single-pool Kt/V; PhA, phase angle; BMI, body mass index; SMI, skeletal muscle index; FFM, fat free mass; TBW, total body water; ECW/TBW, extracellular water/total body water; BFM, body fat mass.

**Table 8:** The association between PhA and depression in MHD patients based on gender difference using binary logistic regression analysis.

Gender	Group	Model 1		Model 2		Model 3		Model 4	
		OR (95% *CI*)	*P*-value	OR (95% *CI*)	*P*-value	OR (95% *CI*)	*P*-value	OR (95% *CI*)	*P*-value
Males	Q1: 2.3°–4.1°	4.400 (1.436–13.479)	0.009	3.645 (1.142–11.639)	0.029	8.748 (1.702–44.951)	0.009	10.363 (0.307–349.731)	0.193
	Q2: 4.2°–4.7°	3.771 (1.195–11.900)	0.024	3.233 (0.997–10.484)	0.051	7.068 (1.524–32.768)	0.012	9.080 (0.718–114.881)	0.088
	Q3: 4.8°–5.4°	0.861 (0.196–3.782)	0.843	0.797 (0.179–3.545)	0.766	1.397 (0.242–8.065)	0.708	1.467 (0.148–14.570)	0.743
Females	Q1: 2.3°–4.1°	11.613 (1.435–93.951)	0.022	16.735 (1.940–144.350)	0.010	31.257 (2.613–373.913)	0.007	80.724 (4.488–859.044)	0.008
	Q2: 4.2°–4.7°	4.324 (0.054–37.081)	0.182	5.067 (0.573–44.832)	0.145	7.903 (0.685–91.213)	0.098	27.852 (1.128–687.789)	0.042
	Q3: 4.8°–5.4°	3.429 (0.385–30.547)	0.270	3.582 (0.396–32.403)	0.256	4.679 (0.395–55.430)	0.221	11.170 (0.648–192.542)	0.097
	Q4: 5.5°–7.4°	1.00	–	1.00	–	1.00	–	1.00	–

Model 1: Unadjusted model.

Model 2: Adjusted age and duration of HD based on Model 1.

Model 3: Adjusted hypertension, diabetes, and dyslipidemia based on Model 2.

Model 4: Adjusted albumin, SMI, FFM, TBW, and ECW/TBW based on Model 3.

## 4 Discussion

This cross-sectional study investigates the relationship between BIA-derived PhA and depressive symptoms in 369 patients receiving MHD. These results robustly demonstrated that lower PhA, a non-invasive marker reflecting cellular integrity and nutritional status, was strongly correlated with higher severity of depressive symptoms, as assessed by the PHQ-9. This relationship persisted as a significant independent predictor even after adjusting for relevant demographic and clinical covariates in multivariate logistic regression models. These findings provide important insights into understanding the potential use of PhA as a predictive marker for mental health in this population.

Depressive symptoms are one of the most common psychological problems in MHD patients. These patients receiving hemodialysis and suffering from depression usually exhibit a variety of adverse clinical outcomes, such as poor nutritional status, cognitive function health, treatment adherence, quality of life, and high mortality rates. In our study, the prevalence of depressive symptoms in MHD patients was 17.10% based on the PHQ-9 score ≥ 10, which relatively aligned with the previous studies. Many studies have reported that the prevalence of depression in dialysis population ranges from 20 to 30% (22). Mapes et al. (23) and Li et al. (8) reported that the prevalences of depression in MHD patients were 20 and 15.2%, respectively. Another study also reported that the prevalence of depressive symptoms in elderly patients with CKD was 23.0% using the 15-item Geriatric Depression Scale (GDS-15) (24). There are significant disparities in the prevalence of depression in different literatures, mainly due to some factors such as the sample size, geographical location, dialysis modality, assessment tool of screening depression, medication use, clinical complications, underlying health conditions, and access to health insurance.

So far, there are limited literatures reporting on the relationship between PhA and depressive symptoms of MHD patients in a Chinese patient population. This study demonstrated that the depressed group exhibited markedly lower mean PhA values with higher PHQ-9 scores, aligning with prior research linking reduced PhA to malnutrition, muscle wasting, sarcopenia, and increased mortality risk in CKD populations (25–27). This finding suggests that compromised cellular health and diminished cell membrane integrity are integral features associated with depression in MHD patients. Noteworthy, the analysis extended beyond PhA, revealing that depression was also associated with significantly poorer body composition profiles. Depressed patients displayed lower SMI, FFM, TBW, and a higher ECW/TBW ratio (8). This constellation of findings—reduced muscle mass, altered fluid distribution favoring extracellular expansion—reinforces the concept of a profound physiological derangement accompanying depression in this cohort. Such fluid imbalance (elevated ECW/TBW) likely exacerbates common MHD complications like edema and contributes to the significant physical burden experienced by these patients, potentially worsening their psychological state (12, 28).

Furthermore, after adjustment of other confounders, PhA values still exhibited independently and significantly associated with depression in MHD patients. PhA has recently gained recognition as a useful biomarker for tracking disease progression and forecasting clinical outcomes in various medical conditions (14, 29). Reduced PhA values suggest compromised cell membrane integrity or enhanced cellular apoptosis, while elevated values are associated with a higher proportion of intact and functional cell membranes (13). The potential pathophysiological mechanisms between PhA and depressive symptoms in MHD patients might be attributed to several factors, including nutritional status, inflammation, oxidative stress, cellular integrity, and immune function. (1) Nutritional status: PhA reflects BCM and nutritional status of patients. Poor nutrition, common in MHD patients, can impair neurotransmitter production, like serotonin and dopamine, which are essential for mood regulation. Deficiencies in these neurotransmitters can contribute to the onset or worsening of depressive symptoms. Lower PhA values may indicate poor nutrition, increasing the risk of depression (28, 30). (2) Inflammation: Chronic inflammation is present in both kidney disease and depression. Higher levels of inflammatory markers, like interleukin-6 (IL-6) and C-reactive protein, are linked to depression. PhA has been shown to correlate with the inflammatory status of the body. Inflammatory processes can affect the brain’s neurotransmitter systems, particularly by increasing the production of inflammatory cytokines that can impair serotonin and dopamine function. Lower PhA values are associated with higher systemic inflammation, which can disrupt neurotransmitter function and contribute to depressive symptoms (31). (3) Oxidative stress: In MHD patients, increased oxidative stress from uremic toxins and dialysis can lead to cellular damage, including brain cells, which is associated with depression. PhA reflects cellular integrity, and a lower PhA is often associated with higher oxidative stress and cellular dysfunction, contributing to both physical debilitation and the development of depressive symptoms (32). (4) Cellular and tissue integrity: PhA measures the integrity of cellular membranes. Lower PhA in MHD patients may indicate compromised cellular function, which can impair brain function, leading to mood disturbances and cognitive decline, both of which are linked to depression (12, 13, 33). (5) Immune function: MHD patients often have immune system dysfunction, which can increase the risk of depression. Malnutrition has been demonstrated to be associated to immune function, and lower PhA values may indicate a weakened immune response. This immune dysfunction can contribute to the development of depression through both direct inflammatory pathways and by impairing the brain’s ability to respond to stress (34, 35). Therefore, the relationship between PhA and depressive symptoms in MHD patients reflects a combination of poor nutrition, inflammation, oxidative stress, cellular dysfunction, and immune system imbalance. PhA offers a non-invasive way to identify patients at higher risk for depression, warranting further research into its use for predicting and managing depression in this population.

Additionally, our study confirmed the significant comorbidity burden associated with depression in MHD patients. The depressed group had a higher prevalence of hypertension, diabetes, and dyslipidemia, conditions known to independently impact both physical and mental well-being. Notably, hypertension emerged as a particularly strong independent correlate of depressive symptoms in the multivariate analysis, which was consistent with the previous study. Through a parsimonious structural equation model, Fan et al. demonstrated that hypertension could significantly influence depression in ESRD patients, even after adjusting for predisposing factors such as personality traits, parental attachment, gender, hemodialysis duration, and other medical conditions (36). While hypoalbuminemia, a marker of nutritional compromise and inflammation, was associated with depression in univariate analysis, its significance attenuated in the multivariate model. This suggests that the composite measure of cellular health and body composition captured by PhA may offer a more holistic and robust indicator of depression risk than albumin alone. The observed correlations between higher PhA values and more favorable biochemical profiles (e.g., lower creatinine, BUN, phosphorus) and better fluid balance (lower ECW/TBW ratio) further support the interconnectedness of cellular integrity, physical health parameters, and mental well-being in MHD patients (29). These results reinforce the hypothesis that interventions improving nutritional status and muscle mass, thereby increasing PhA, can yield dual benefits for both physical and mental health outcomes, including potentially mitigating depressive symptoms. Furthermore, our gender-specific analysis underscores the importance of considering gender differences when evaluating the relationship between PhA and depression in MHD patients. Although both genders show a negative association between PhA and depressive symptoms, the strength of the association appears to be stronger in females. This could be attributed to differences in body composition, as reflected in the significant differences in PhA values between genders. Females in this cohort had lower PhA values, which might indicate altered cell membrane integrity, a known indicator of poor nutritional status and a potential risk factor for depression. The results also highlight the complex interplay between clinical parameters, such as ferritin, albumin, and PhA, and depressive symptoms in MHD patients. This analysis reinforces the need to monitor PhA as a potential biomarker for identifying individuals at high risk for depression, particularly in females, where the association appears more pronounced. This finding suggests that gender differences should be considered in the evaluation of PhA values in clinical practice, particularly in chronic hemodialysis patients, where gender-specific intervention strategies may be more effective. Further studies with larger sample sizes are warranted to confirm these findings and explore the underlying mechanisms that contribute to gender differences in the PhA-depression relationship.

Overall, this study underscores the critical importance of integrating mental health screening, particularly for depression, into the routine care of MHD patients. PhA measurement, being simple, rapid, non-invasive, and cost-effective, emerges as a promising tool for identifying patients at heightened risk for these debilitating conditions, such as depression and cognitive dysfunction. Monitoring PhA can provide valuable insights into a patient’s underlying cellular health and nutritional status, prompting timely interventions. Future research should prioritize as follows: (1) Mechanistic exploration: Delineating the precise biological pathways (e.g., oxidative stress, hormonal dysregulation, and inflammation) linking low PhA, altered body composition, fluid imbalance, and depression in MHD patients. (2) Longitudinal studies: Investigating whether changes in PhA over time predict the onset or progression of depressive symptoms. (3) Intervention trials: Evaluating the efficacy of targeted strategies (e.g., nutritional supplementation, exercise programs, combined multimodal approaches) aimed at improving PhA, muscle mass, and nutritional status, and assessing their impact on mental health and cognitive outcomes in this vulnerable population.

## 5 Limitations

This study has some limitations: (1) Cross-sectional design: The study design was cross-sectional, meaning that it only provides a snapshot of the association between PhA values and depressive symptoms at a single point in time. This limits our ability to infer causal relationships between PhA and depression. Longitudinal studies will be needed to establish the directionality and causality of this relationship. (2) Potential confounding factors: Despite adjusting for a wide range of clinical and biochemical factors, there may still be unmeasured confounders that can influence the observed associations. For example, factors such as physical activity levels, dietary intake, or medications (other than those considered in the study) may have an impact on both PhA values and depression but are not included in the analysis. The use of antidepressants and other medications was not considered in this study, and their potential effects on depressive symptoms should be explored in future research. (3) Generalizability: The study focused on MHD patients, a specific subgroup of individuals with chronic kidney disease. Therefore, the findings may not be applicable to the general population or to patients with other types of kidney disease or conditions. The results may also not be generalizable to populations in different geographic regions or healthcare settings. (4) PhA as a surrogate marker: While PhA is a useful tool for assessing cellular integrity and nutritional status, it is an indirect measure and may not fully capture all factors contributing to depressive symptoms. Other biomarkers or diagnostic tests might provide a more comprehensive assessment of the relationship between nutrition, cellular health, and depressive symptoms in MHD patients.

## 6 Conclusion

In conclusion, this study establishes BIA-derived PhA as a significant and independent correlate of depressive symptoms in MHD patients. Lower PhA values, indicative of compromised cellular integrity and poorer nutritional/muscle status, are associated with worse mental health outcomes. These findings highlight that PhA may serve as a useful indicator of depressive symptoms in MHD patients, and use as a valuable, practical biomarker with potential utility in risk stratification and guiding integrated care approaches that address the complex interplay between physical and mental health in the hemodialysis setting. Further studies are needed to elucidate the underlying mechanisms linking PhA to depression, as well as to evaluate the efficacy of targeted interventions aimed at improving nutritional status and muscle mass in this population.

## Data Availability

The raw data supporting the conclusions of this article will be made available by the authors, without undue reservation.
